# Hypertension preserves the magnitude of microvascular flow‐mediated dilation following transient elevation in intraluminal pressure

**DOI:** 10.14814/phy2.14507

**Published:** 2021-02-15

**Authors:** William E. Hughes, Natalya Zinkevich, David D. Gutterman, Andreas M. Beyer

**Affiliations:** ^1^ Department of Medicine Medical College of Wisconsin Milwaukee WI USA; ^2^ Cardiovascular Center Medical College of Wisconsin Milwaukee WI USA; ^3^ Department of Health and Medicine Carroll University Waukesha WI USA; ^4^ Department of Physiology Medical College of Wisconsin Milwaukee WI USA

**Keywords:** flow‐mediated dilation, hypertension, intraluminal pressure

## Abstract

**Objective:**

The objective of this study was to measure flow‐mediated dilation (FMD) prior to and following transient increases in intraluminal pressure (IILP) in resistance arterioles isolated from subjects with and without coronary artery disease (CAD) (CAD and non‐CAD) and non‐CAD subjects with hypertension.

**Methods:**

Arterioles were isolated from discarded surgical tissues (adipose and atrial) from patients without coronary artery disease (non‐CAD; ≤1 risk factor, excluding hypertension), with CAD, and non‐CAD patients with hypertension (hypertension as the only risk factor). To simulate transient hypertension, increased IILP was generated (150 mmHg, 30 min) by gravity. Arterioles were constricted with endothelin‐1, followed by FMD and endothelial‐independent dilation prior to and following exposure to IILP.

**Results:**

IILP reduced FMD in non‐CAD and CAD arterioles relative to pre‐IILP (*p* <.05 at 100 cmH_2_O). In contrast, arterioles from non‐CAD hypertensive subjects exhibited no reduction in maximal FMD following IILP (*p* = .84 at 100 cmH_2_O). FMD was reduced by L‐NAME prior to IILP in non‐CAD hypertensive patients (*p* < .05 at 100 cmH_2_O); however, following IILP, FMD was inhibited by peg‐cat (*p* < .05 at 100 cmH_2_O), indicating a switch from NO to H_2_O_2_ as the mechanism of dilation.

**Conclusions:**

Acute exposure (30 min) to IILP (150 mmHg) attenuates the magnitude of FMD in non‐CAD and CAD resistance arterioles. The presence of clinically diagnosed hypertension in non‐CAD resistance arterioles preserves the magnitude of FMD following IILP as a result of a compensatory switch from NO to H_2_O_2_ as the mechanism of dilation.

**New and Noteworthy**

Exposure to acute pressure stress reduces magnitude of flow‐mediated dilation (FMD) in arterioles from non‐coronary artery disease (CAD; ≤1 cardiovascular risk factor, excluding hypertension) and CAD patients. This reduction is also associated with a switch from nitric oxide to hydrogen peroxide in non‐CAD. We demonstrate that presence of clinical hypertension preserves the magnitude of FMD through a compensatory switch to hydrogen peroxide, perhaps “pre‐conditioning” the microvasculature to protect against pressure insults.

## 1 INTRODUCTION

Endothelium‐dependent dilation regulates blood flow, and integrity of endothelial‐dependent dilation is a barometer of vascular health and propensity for cardiovascular events (Targonski et al., 2003). Flow‐mediated dilation (FMD) is a physiological response to shear stress along the endothelium, and an index of overall endothelial health and function. Dysfunction of the microvascular endothelium (e.g., endothelial dysfunction), particularly within the coronary resistance arterioles, predisposes cardiac tissue to ischemia and atherosclerosis, making the treatment of endothelial dysfunction an attractive pharmacological target (Daiber et al., 2017). Under physiological conditions, the primary mediator of microvascular FMD in humans is nitric oxide (NO); however, with the development of coronary artery disease (CAD) or acute exposure (30 min) to increased intraluminal pressure (IILP; 150 mmHg/204 cmH_2_O) in arterioles from non‐CAD patients (no evidence of CAD and ≤1 cardiovascular risk factor), the mechanism of microvascular vasodilation switches to hydrogen peroxide (H_2_O_2_) (Beyer et al., 2017). The switch in the primary mechanism of FMD with CAD or high pressure stress is compensatory and associated with reductions in NO bioavailability concomitant to elevations in mitochondria‐derived reactive oxygen species (Beyer et al., 2014; Targonski et al., 2003). In subjects with CAD, who already rely on the compensatory dilation to H_2_O_2_, FMD is mostly eliminated after IILP (Kadlec et al., 2017). It is not known if the presence of key risk factors for CAD (e.g., hypertension) influences the ability of the microcirculation to compensate for stress‐induced loss of NO‐mediated dilation to flow.

Hypertension is a key clinical risk factor in the development of CAD, and is associated with elevated ROS and reduced NO bioavailability (Taniyama & Griendling, 2003). Patients with hypertension experience prolonged elevations in blood pressure, often coinciding with endothelial dysfunction (Dharmashankar & Widlansky, 2010; Higashi, Kihara, & Noma, 2012). It is unknown whether hypertension alone in patients without CAD results in similar reductions in the magnitude of microvascular FMD following acute exposure to IILP. Our lab has previously demonstrated that acute elevations in blood pressure via lower body isometric exercise reduce acetylcholine (ACh)‐induced dilation in arterioles from sedentary individuals, while arterioles from chronically exercise‐trained individuals maintain dilation to ACh, but rely on a compensatory switch to H_2_O_2_ as the mediator of dilation (Durand et al., 2015). Whether this “protective” effect of intermittent hypertension is a local vascular phenomenon or due to circulating systemic factors is not known. The goal of this study was to determine if fundamental vascular changes within subjects with hypertension are responsible for the maintained endothelium‐dependent dilation. It has previously been shown that intermittent exposure to physiological high pressure may precondition the vasculature against acute high pressure insults (Durand et al., 2015; Phillips, Das, Wang, Pritchard, & Gutterman, 2011); however, whether pathological chronic increases in blood pressure elicit similar responses is unknown.

We hypothesized that an increase in IILP in isolated resistance arterioles taken from patients with clinically diagnosed hypertension would demonstrate a preserved FMD magnitude as a result of a vascular adaptation and not due to neurohumoral factors acting on the arterioles.

## 2 MATERIALS AND METHODS

### 2.1 Tissue acquisition and general protocol

All protocols were approved by the Institutional Review Board of the Medical College of Wisconsin. Discarded subcutaneous, visceral, or pericardial adipose and atrial appendages were obtained from the MCW Tissue Bank at the time of surgery. Whole hearts, not used for transplantation or valve harvesting, were obtained from the Wisconsin Donor Network organ procurement and placed in ice cold (4°C) HEPES buffer solution. All tissues were de‐identified and considered as surgical discard. Insufficient number of samples were collected to stratify by race or sex. Arterioles were cleaned of fat and connective tissue, cannulated in a tissue bath, and were prepared for continuous measurement of diameter. Delineation of groups was based on the presence of cardiovascular risk factors. Healthy tissue (non‐CAD) was defined as having one or less cardiovascular risk factor, excluding hypertension. The hypertensive group was classified as non‐CAD having hypertension alone as a risk factor. CAD was defined as having a clinical diagnosis of CAD. We have previously published non‐CAD (≤1 risk factor excluding hypertension) and CAD data pre‐ and post‐IILP (Beyer et al., 2014; Kadlec et al., 2017). FMD data within these groups pre‐ and post‐IILP are used within this manuscript for comparison purposes only.

### 2.2 Materials

Endothelin‐1 (ET‐1) was obtained from Peninsula Laboratories. Other chemicals were obtained from Sigma‐Aldrich. ET‐1 was prepared in saline with 1% bovine serum albumin. N^G^‐nitro‐L‐arginine methyl ester (L‐NAME; 100 µM), polyethylene glycol catalase (peg‐cat; 500 U/ml), rotenone (1 µM), and 2‐(4‐carboxyphenyl)‐4,4,5,5‐tetramethylimidazoline‐1‐oxyl‐3‐oxide (c‐PTIO; 100 µM) were used to inhibit vasodilator pathways to elucidate the mechanism of dilation pre‐ and post‐IILP similar to our previous publications (Beyer et al., 2014; Kadlec et al., 2017). ET‐1 was used to preconstrict arterioles, L‐NAME inhibits endothelial nitric oxide synthase, peg‐cat acts as a H_2_O_2_ scavenger, rotenone is a mitochondrial complex I inhibitor, and c‐PTIO scavenges NO. Agents were prepared in distilled water or physiological saline solution. All concentrations are expressed as the final steady‐state concentrations in the tissue bath.

#### 2.2.1 Cannulated vessel preparation

Vasodilator responses to physiological flow in arterioles were evaluated as previously described by our lab (Miura, Liu, & Gutterman, 1999). Briefly, in an organ chamber, resistance arterioles were cannulated with Krebs buffer‐filled glass micropipettes of matching impedance, and pressurized (60 mmHg) for continuous video microscopy of vessel diameter changes. Vessels were preconstricted with ET‐1 (0.1–1 nM) to achieve a 20%–50% stable reduction in passive diameter. Flow was induced through the cannulated arterioles using glass micropipettes by changing the heights of two Krebs buffer‐filled reservoirs in equal but opposite directions. Pressure‐induced flow gradients of 5–100 cmH_2_O were generated and steady‐state diameter was measured at 5 min following each change in pressure. This range of pressure gradients using matched pipettes of minimal internal diameter of ~40 µm produces a calculated shear stress in the physiological range of between 5 and 30 dynes/cm^2^. Flow‐response curves were generated for each vessel prior to and following elevations in IILP (150 mmHg/204 cmH_2_O for 30 min), as previously published, either alone or in the presence of various inhibitors (Beyer et al., 2014). Two flow‐response curves were generated for each vessel, one before and one after the 30‐min IILP. When possible, two vessels from the same tissue were used, with and without pharmacological intervention (see above). Data are reported as the percent of maximal change in diameter for a given flow gradient.

### 2.3 Statistical methods

Data are presented as mean ± *SD* for percent dilation in response to flow (endothelial‐dependent) or papaverine (endothelial‐independent). Non‐CAD (≤1 risk factor excluding hypertension) and CAD data have been previously published, and are used for subjective comparison only. Given that the non‐CAD and CAD data have been previously published, an a priori sample size was not calculated. A two‐way ANOVA was used with flow gradient and inhibitors as parameters. When a significant difference was observed, responses at individual flow gradients were compared using a Holm–Sidak multiple comparison test. Post hoc effect sizes were evaluated at 100 cmH_2_O using Cohen's *d*. Statistical significance was determined at a *p *< .05.

## 3 RESULTS

Patient characteristics are shown in Table 1. Prior to preconstriction, baseline internal diameters were not different pre‐versus post‐IILP (*p* = .99 for all). There were no differences within groups pre‐versus post‐IILP for percent constriction to ET‐1 (Table 1). The average maximal vascular internal diameters were not different following IILP in any group (*p* = .99 for all).

**Table 1:** Patient characteristics

	Non‐CAD + HTN	Historical Non‐CAD	Historical CAD
Total samples (*n*)	11	5	5
Sex (M/F)	4/7	2/3	4/1
Age (years)	56 ± 15	43 ± 14	73 ± 9
Race (AA/Cauc/Hisp/Asian)	3/8/0/0	0/5/0/0	0/5/0/0
Underlying risk factors			
Current tobacco use	0	1	1
Hypertension	11	0	4
Hypercholesterolemia	0	1	5
No risk factors	0	3	0
Underlying diseases			
Coronary artery disease	0	0	5
Congestive heart failure	0	0	0
Myocardial infarction	0	0	1
Vessel characteristics			
Baseline Diameter (µm)			
Pre‐IILP	158 ± 50	182 ± 78	77 ± 8
Post‐IILP	185 ± 75	195 ± 87	71 ± 12
Percent constriction (%)			
Pre‐IILP	33 ± 9	36 ± 17	44 ± 9
Post‐IILP	38 ± 11	32 ± 10	35 ± 16
Maximal internal diameter (µm)			
Pre‐IILP	211 ± 75	233 ± 95	114 ± 10
Post‐ IILP	237 ± 90	239 ± 108	111 ± 25

Note

Mean ± *SD*.

Abbreviations: AA, African‐American; CAD, coronary artery disease; Cauc, Caucasian; HTN, hypertension; Hisp, Hispanic; IILP, increased intraluminal pressure.

### 3.1 Effect of increased IILP on FMD

**Figure 1:**
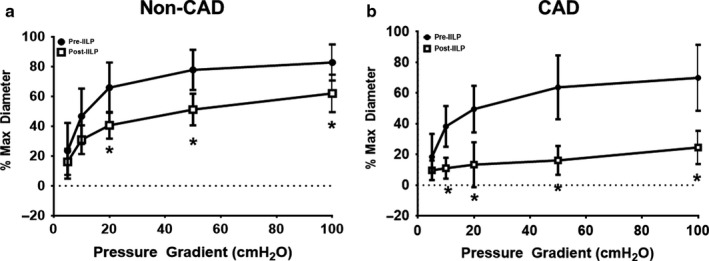
Historical flow‐mediated dilation (FMD) responses in non‐CAD (a) and CAD (b) patients prior to and following exposure to acute (30 min) increased intraluminal pressure (IILP; 150 mmHg). FMD magnitude is decreased in both resistance arterioles from non‐CAD (≤1 risk factor) and CAD patients. These data are adapted from previous publications (Beyer et al., 2014; Kadlec et al., 2017). *p < .05 versus pre‐IILP

**Figure 2:**
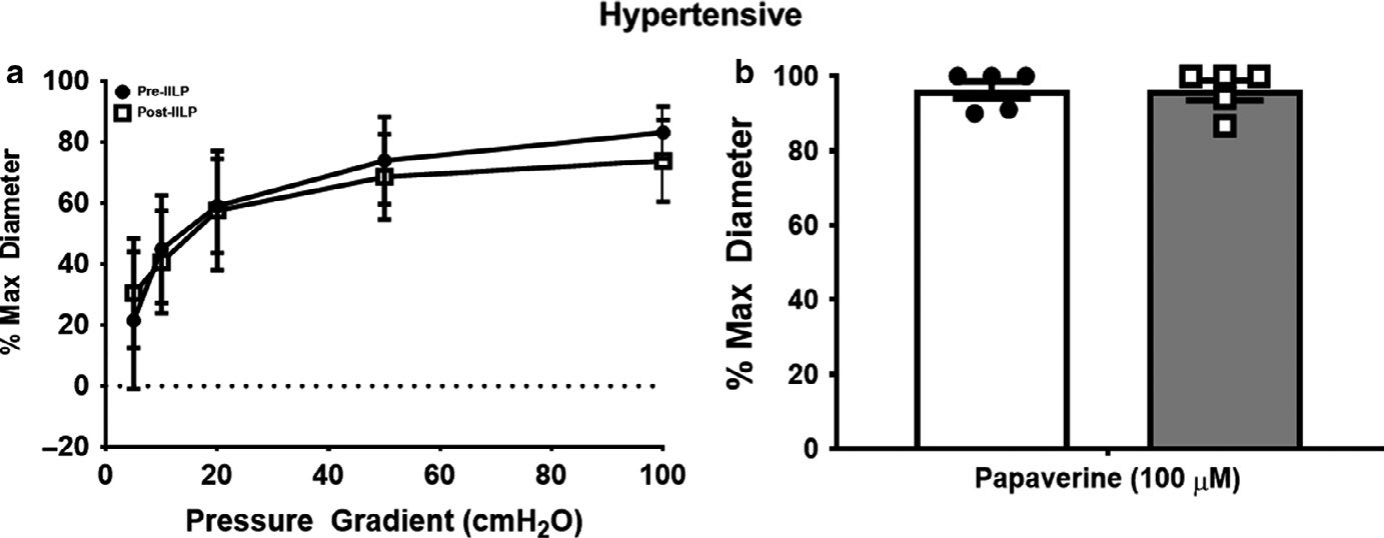
Flow‐mediated dilation (FMD; a) and endothelial‐independent (b) responses in non‐CAD hypertensive resistance arterioles prior to and following exposure to acute (30 min) increased intraluminal pressure (IILP; 150 mmHg). FMD magnitude is preserved in non‐CAD hypertensive resistance arterioles post‐IILP

Based on previously published experiments, exposure to 30 min of IILP reduces FMD magnitude in arterioles from non‐CAD (≤1 risk factor excluding hypertension) and CAD patients (range: 24%–34% and 70%–87% reduction, respectively) relative to pre‐IILP (Fig. 1 in Beyer et al., 2014 and Fig. 5a in Kadlec et al., 2017, respectively). This previously published data are summarized in Figure 1a,b. In contrast, resistance arterioles from non‐CAD patients with hypertension exhibited preserved magnitude of FMD in response to IILP (Figure 2a; Cohen's *d* at 100 cmH_2_O = 0.64).

### 3.2 Effect of Increased IILP on the Mechanism of Dilation in Subjects with Clinical Hypertension

**Figure 3:**
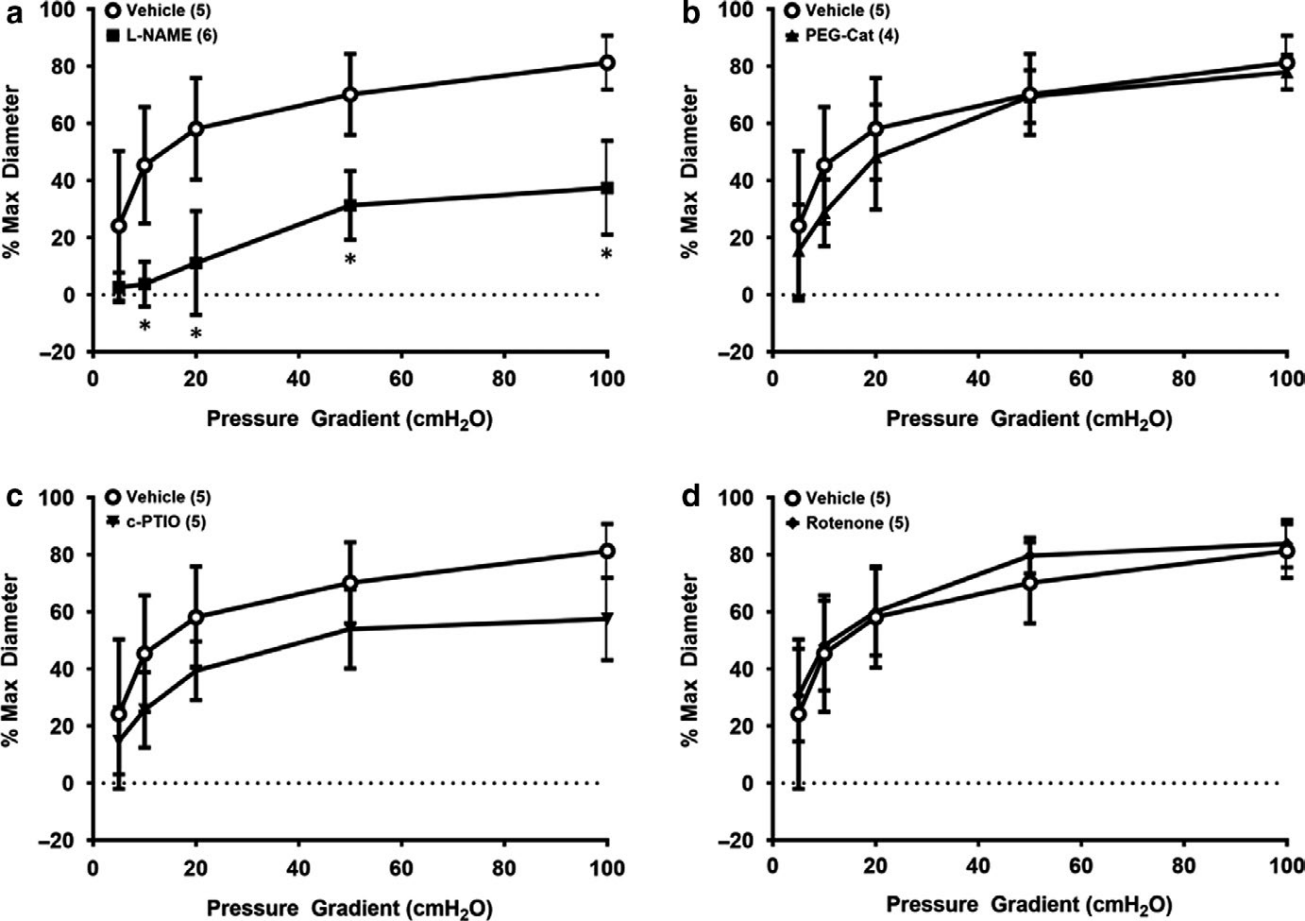
Mechanism of flow‐mediated dilation in non‐CAD hypertensive resistance arterioles prior to acute (30 min) increased intraluminal pressure (IILP; 150 mmHg). Prior to IILP exposure, L‐NAME reduces the magnitude of dilation indicating NO synthase as the predominant mechanism of dilation (a), while peg‐cat (b), c‐PTIO (c), and rotenone (d) do not reduce FMD. *p < .05 L‐NAME versus vehicle

**Figure 4:**
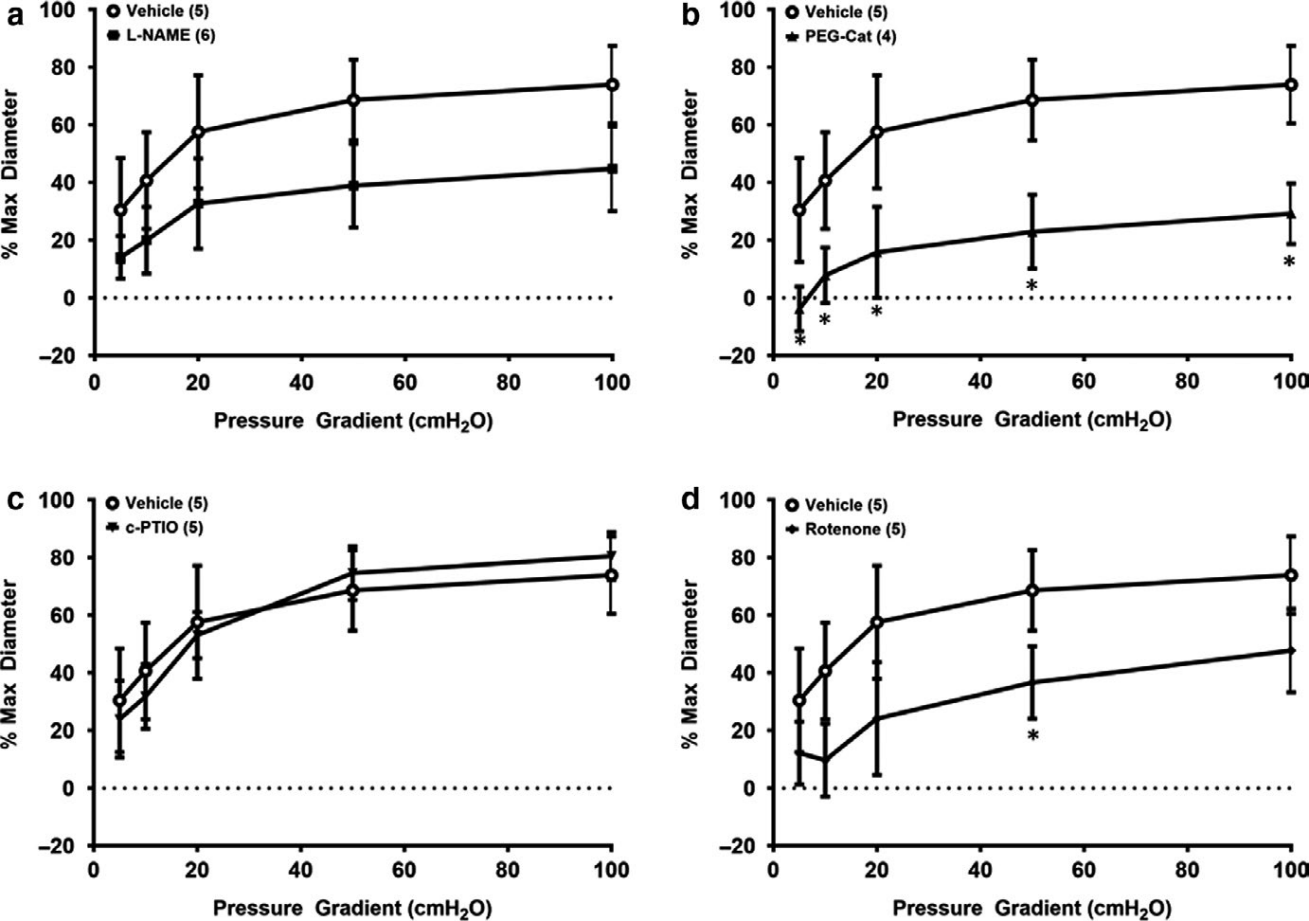
Mechanism of flow‐mediated dilation in non‐CAD hypertensive resistance arterioles following exposure to acute (30 min) increased intraluminal pressure (IILP; 150 mmHg). Following IILP, peg‐cat reduces dilation following exposure to IILP (b), while L‐NAME (a) and c‐PTIO (c) do not reduce FMD. Rotenone (d) reduced FMD magnitude at 50 cmH_2_O only. *p < .05 Peg‐Cat (a) versus vehicle; #*p* < .05 for rotenone versus vehicle (e)

In isolated vessels form subjects with clinical hypertension, the mechanism of dilation is mediated by NO synthase as demonstrated by a reduction in FMD magnitude in the presence of L‐NAME (Figure 3a; *p* < .05; Cohen's *d* at 100 cmH_2_O = 3.27). However, no reductions in FMD in the presence of peg‐catalase (Figure 3b; *p* = .60–.99; Cohen's *d* at 100 cmH_2_O = 0.41), c‐PTIO (Figure 3c; *p* = .16–.97; Cohen's *d* at 100 cmH_2_O = 1.96), or rotenone (Figure 3d; *p* = .73–.99; Cohen's *d* at 100 cmH_2_O = 0.27) were observed prior to IILP exposure. Following acute exposure to IILP, a switch in the mechanism of dilation to predominantly H_2_O_2_ was detected as evidenced by a reduction in FMD magnitude in the presence of peg‐catalase (Figure 4b; *p* < .05; Cohen's *d* at 100 cmH_2_O = 3.7), but not L‐NAME (Figure 4a; *p* = .09–.46; Cohen's *d* at 100 cmH_2_O = 2.1) or c‐PTIO (Figure 4c; *p* = .83–.89; Cohen's *d* at 100 cmH_2_O = 0.67). The mitochondrial complex I inhibitor rotenone reduced dilation at flow 50 cmH_2_O post‐IILP (Figure 4d; *p* < .05; Cohen's *d* at 100 cmH_2_O = 0.88). Endothelial‐independent dilation was not altered in response to IILP in any group (Table 1, and Beyer et al., 2014; Kadlec et al., 2017).

## 4 DISCUSSION

The objective of this study was to determine if the presence of chronic hypertension influenced the effects of acute exposure to high IILP on endothelial‐dependent vasodilation in resistance arterioles. There are two novel findings of this study. First, the magnitude of arteriolar FMD following acute exposure to pressure stress is maintained in patients with hypertension in contrast to our prior observation that IILP reduces FMD in vessels from patients without hypertension or those with CAD. Second, this preservation of FMD magnitude appears to be the result of a compensatory switch in the mechanism of dilation from primarily NO synthase‐mediated dilation pre‐IILP to one that is mostly mediated by mitochondrial H_2_O_2._

Our previous work indicates that arterioles from non‐CAD patients (≤1 risk factor and excluding hypertension) demonstrate a reduction in magnitude of dilation to shear stress (Beyer et al., 2014) and ACh (Durand, Phillips, Widlansky, Otterson, & Gutterman, 2014), which is coupled with a switch in the mechanism of dilation from NO to H_2_O_2_ in response to acute IILP. These pressure‐induced reductions in FMD and ACh dilation were associated with an increase in mitochondria‐derived H_2_O_2_ production and were prevented by the administration of sepiapterin (FMD) (Beyer et al., 2014; Durand et al., 2014). The current study extends upon these findings, demonstrating that in arterioles from non‐CAD patients with only hypertension as a risk factor, FMD magnitude following IILP is preserved.

Interestingly, c‐PTIO had no effect on FMD magnitude prior or post‐IILP exposure in arterioles from hypertensive non‐CAD patients (Figure 3c).This contrasts with our previous findings in subjects without CAD or hypertension where scavenging of NO with c‐PTIO reduced FMD (Beyer et al., 2014).This finding suggests a NO synthase‐dependent, but NO‐independent dilation occurs in subjects with hypertension. Peg‐catalase did not reduce FMD magnitude in arterioles from non‐CAD hypertensive patients, therefore it is unlikely that the mediator of dilation is uncoupled endothelial NO synthase. Previous reports demonstrate that citrulline, a precursor for arginine synthesis, and thereby NO production by endothelial NO synthase exert some vasodilator effects; however, this is controversial (Allerton et al., 2008; Churchward‐Venne et al., 2014; Gonzales, Raymond, Ashley, & Kim, 2017; Xuan et al., 2015). This would explain the reduction in FMD magnitude with L‐NAME, yet lack of effect of c‐PTIO. Identification of the mechanism of dilation in subjects with hypertension represents an intriguing area for future investigation.

Hypertension is a clinical risk factor in the development of cardiovascular disease (Franklin & Wong, 2013), and is associated with reductions in endothelial‐dependent dilation (ACh) in both conduit and resistance arteries (Egashira et al., 1995; Endemann et al., 2004; Millgard & Lind, 1998; Panza, Quyyumi, Brush, & Epstein, 1990; Rizzoni et al., 1998; Shimbo et al., 2010). Data from other labs have demonstrated that brief 1 or 4 hr of exposure to high pressure (~95 mmHg) in rat soleus feed arteries improved FMD, and ACh‐induced vasodilation in older rats (no improvement in young), and these responses were inhibited partially by L‐NAME, and more completely by combined inhibition of L‐NAME and indomethacin (Seawright, Luttrell, Trache, & Woodman, 2016; Seawright, Luttrell, & Woodman, 2014; Woodman, Trott, & Laughlin, 2007). Data from our lab indicate that acute elevations in blood pressure as a result of lower body exercise or IILP attenuate microvascular endothelial‐dependent dilation (ACh) in sedentary individuals, while in regularly exercising subjects, endothelial‐dependent dilation was maintained following pressure stress through a compensatory switch from NO/cyclooxygenase to H_2_O_2_ (Durand et al., 2015). These findings lend credence that physiological increases in blood pressure elicit change in vascular function, such that chronic, intermittent exposure to elevated blood pressure (e.g., exercise) confers protection against acute elevated pressure insults. Given these previous findings from both animal models and humans and current findings, it would be interesting to speculate that both physiological (exercise) and pathological chronic exposure to high pressure (e.g., hypertension) “conditions” arterioles to minimize the detrimental vasomotor impact of the stress of transient increases in IILP. This plasticity in vasomotor responsiveness requires further investigation.

We have previously demonstrated that arterioles from CAD patients exhibit a compensatory vasodilator response to shear stress, such that they rely primarily on mitochondria‐derived H_2_O_2_ (Beyer et al., 2017; Phillips, Hatoum, & Gutterman, 2007) Kadlec et al. (2017) demonstrated that acute IILP drastically reduces FMD magnitude in CAD patients, and upregulation of PGC1α ameliorated this response. Interestingly, four of the five CAD patients had a diagnosis of hypertension yet each demonstrated a blunted FMD magnitude (57%–65%) post‐IILP. We hypothesize that as arterioles from CAD patients already demonstrate a compensatory dilator mechanism (H_2_O_2_), the added burden of IILP (which switches the mechanism from NO to H_2_O_2_ in arterioles from non‐CAD patients) acts as a “double hit” resulting in a frank loss of dilation, or perhaps a constrictor response.

### 4.1 Experimental considerations

There are a few experimental considerations within the present study. First, we are unable to provide insight into the attenuation of FMD in arterioles from patients with CAD in response to IILP. However, as discussed, the magnitude of FMD in arterioles from CAD patients is dramatically reduced such that the remaining dilation is trivial, making the mechanism of dilation mostly irrelevant. Second, patient information was obtained from a one page questionnaire and not the patient's chart (these were discarded anonymous surgical specimens), and limited medical history is available including medications which could have influenced our results. Given the nature of experiments utilizing surgical discard tissue, it is difficult to adequately match for sex, age, and race. Additionally, we did not select vessels based on size for experiments; however, resistance arterioles examined within our lab generally range between 100 and 150 µm. As we have primarily used historical data for comparison, an a priori sample size was not calculated. Prior to physiological measurements, vessels were thoroughly washed with buffer solution, thus residual effects of any systemic medications were likely minimal. Finally, the duration of hypertension in these patients is not known and could have influenced the results.

## 5 CONCLUSIONS

This is the first study to demonstrate the preservation of FMD in arterioles exposed to high pressure in subjects with underlying hypertension. The preserved dilation was mediated by NO synthase before and H_2_O_2_ after exposure to IILP. Our previous work demonstrates that IILP reduces FMD in patients with CAD or in those without CAD or HTN. When taken in context with previously published reports, these results suggest that high pressure exposure “precondition” the microvasculature of hypertensive subjects, protecting the microvasculature against acute pressure insults through compensatory mechanisms.

## 6 ETHICS

All tissues used for this study were obtained as otherwise de‐identified surgical discard tissues as approved under MCW IRB# PRO00000114 and PRO00001094.

## DISCLOSURES

The authors have no conflict of interest or disclosures.

## AUTHOR CONTRIBUTION

WEH, analyzed and interpreted data, drafted, and critically revised manuscript; NZ, collected data, and critically revised manuscript; DDG, contributed to study design and critically revised manuscript; AB, contributed to study design, collected data, and critically revised manuscript.
